# A novel wearable device integrating ECG and PCG for cardiac health monitoring

**DOI:** 10.1038/s41378-024-00858-3

**Published:** 2025-01-15

**Authors:** Junbin Zang, Qi An, Bo Li, Zhidong Zhang, Libo Gao, Chenyang Xue

**Affiliations:** 1 College of Information Engineering, Shanxi College of Technology, Shuozhou, 036000 China; 2 Key Laboratory of Instrumentation Science and Dynamic Measurement Ministry of Education, North University of China, 030051 Taiyuan, China; 3 Department of Mechanical and Electrical Engineering, Xiamen University, 361102 Xiamen, China

**Keywords:** Electrical and electronic engineering, Materials science

## Abstract

The alarming prevalence and mortality rates associated with cardiovascular diseases have emphasized the urgency for innovative detection solutions. Traditional methods, often costly, bulky, and prone to subjectivity, fall short of meeting the need for daily monitoring. Digital and portable wearable monitoring devices have emerged as a promising research frontier. This study introduces a wearable system that integrates electrocardiogram (ECG) and phonocardiogram (PCG) detection. By ingeniously pairing a contact-type PZT heart sound sensing structure with ECG electrodes, the system achieves the acquisition of high-quality ECG and PCG signals. Notably, the signal-to-noise ratios (SNR) for ECG and PCG signals were measured at 44.13 dB and 30.04 dB, respectively, demonstrating the system’s remarkable stability across varying conditions. These collected signals were subsequently utilized to derive crucial feature values, including electromechanical delay (EMD), left ventricular ejection time (LVET), and pre-ejection period (PEP). Furthermore, we collected a dataset comprising 40 cases of ECG and PCG signals, enabling a comparative analysis of these three feature parameters between healthy individuals and coronary heart disease patients. This research endeavor presents a significant step forward in the realm of early, non-invasive, and intelligent monitoring of cardiovascular diseases, offering hope for earlier detection and more effective management of these life-threatening conditions.

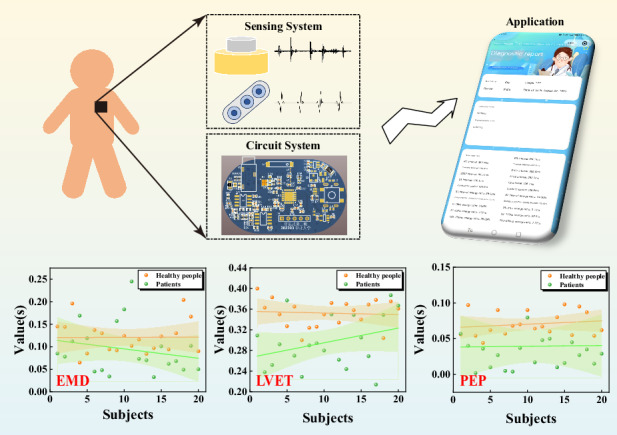

## Introduction

According to a recent report by the World Health Organization, the prevalence of cardiovascular diseases is steadily on the rise^1^, posing a formidable threat to human mortality^2^. These diseases are often labeled as “silent killers” due to their tendency to exhibit minimal or no discernible symptoms during their initial stages^3^. Nevertheless, research has demonstrated that early interventions can significantly lower mortality rates among those affected. ECG and PCG signals represent crucial physiological indicators of cardiac health^4^, each offering unique insights into the heart’s functioning. ECG signals capture the electrical activities of the heart, while PCG signals reflect its mechanical vibrations^5–7^. The combined analysis of these two signals has proven to be a powerful tool in enhancing the accuracy and efficiency of early cardiovascular disease diagnosis^8^.

Heart sound auscultation, an age-old diagnostic technique, has stood the test of time through continuous refinement and adaptation^9^. Similarly, ECG signals have traditionally been captured using electrocardiograph machines. However, with the relentless pace of technological advancement, the limitations of these traditional methods have become increasingly apparent, failing to keep up with the demands of modern healthcare. In light of this, the development of a novel digital, portable, and wearable device capable of simultaneously detecting both ECG and PCG signals holds immense clinical and societal significance.

Currently, numerous scholars are actively engaged in digital research about heart sound sensors. This includes individuals such as Chenzheng Zhou^10^, Yuhua Yang^11^, Qi An^12^, and many others who have made significant contributions to the field. These researchers have leveraged bionic principles to develop a diverse array of MEMS piezoresistive heart sound sensing structures, aimed at advancing cardiac health monitoring capabilities. Furthermore, Mengjiao Qu and her team^13^ have introduced a pioneering MEMS AlN heart sound sensor structure, while Zhenghao Chen and his colleagues^14^ have employed piezoelectric elements to capture intricate human heart sound signals. Flexible sensing technology has also been successfully applied to heart sound detection by Boyan Liu^15^, Yi Luo^16^, and their respective teams, enabling the effective capture of heart sound signals. Additionally, Ken Ogawa and his collaborators^17^ have pioneered the use of single-fiber Bragg grating (FBG) sensors for the simultaneous detection of human heart sounds, pulses, and respiration, further expanding the scope of heart sound monitoring. Researchers like Guo Binbin^18^ and Tian Wang^19^ have gone a step further, proposing array-type heart sound sensors that offer a more comprehensive and nuanced approach to heart sound signal detection.

Despite the notable strides made in the realm of digital heart sound sensors, there remains a dearth of research focused on the synchronous detection of ECG and PCG signals. Sofia M. Monteiro and her colleagues^20^ made a valuable contribution by employing electret microphones and polymer dry electrodes to detect both ECG and PCG signals. However, Pengcheng Shi and his team^21^, while proposing an innovative integrated ECG-PCG synchronous device utilizing MEMS bionic heart sound sensors, faced a limitation in their design: the use of lead wires for the electrocardiogram component, rendering it unsuitable for portable and wearable applications.

More recently, Cho, Han Seok, and their team^22^ have introduced a wearable device capable of synchronously capturing ECG and PCG signals. While this represents a significant advancement, it still relies on electret microphone heart sound sensors, potentially limiting its overall performance and versatility.

The primary objective of this study is to design and develop a non-invasive, wearable, cost-effective, and user-friendly device that integrates ECG and PCG detection capabilities. This innovative device is not only highly sensitive and portable but also easy to manufacture, making it an ideal solution for a wide range of users. Furthermore, this research endeavor seeks to pioneer a novel signal recognition method that significantly enhances early diagnosis and prevention capabilities for cardiovascular diseases. By leveraging this advanced methodology, we aim to reduce detection costs, ultimately contributing to the overall well-being of individuals and communities. The key contributions of this research work can be summarized as follows:

(a) We propose a novel contact-type heart sound sensor structure based on PZT sensors, which offers unparalleled sensitivity and accuracy in detecting heart sound signals.

(b) We establish a portable wearable electrocardiogram and heart sound synchronous acquisition system, enabling real-time monitoring of both ECG and PCG signals conveniently.

(c) We conduct a comprehensive analysis of ECG and PCG signals from both healthy individuals and patients, focusing on three crucial characteristic parameters: EMD, LVET, and PEP. This analysis provides valuable insights into the relationship between these parameters and cardiovascular health, ultimately contributing to the development of more effective diagnostic and prevention strategies.

The organization of this paper is structured as follows: in the first chapter, we provide an in-depth overview of the background and current research status of ECG and PCG detection technology. The second chapter delves into the design principles and implementation methods of the wearable ECG and PCG detection device that we have developed in this study. In the third chapter, we analyze and discuss the performance of the sensors that comprise our device. Finally, the fourth chapter summarizes the key research findings of our study and outlines potential future research directions.

## Materials and methods

**Fig. 1:**
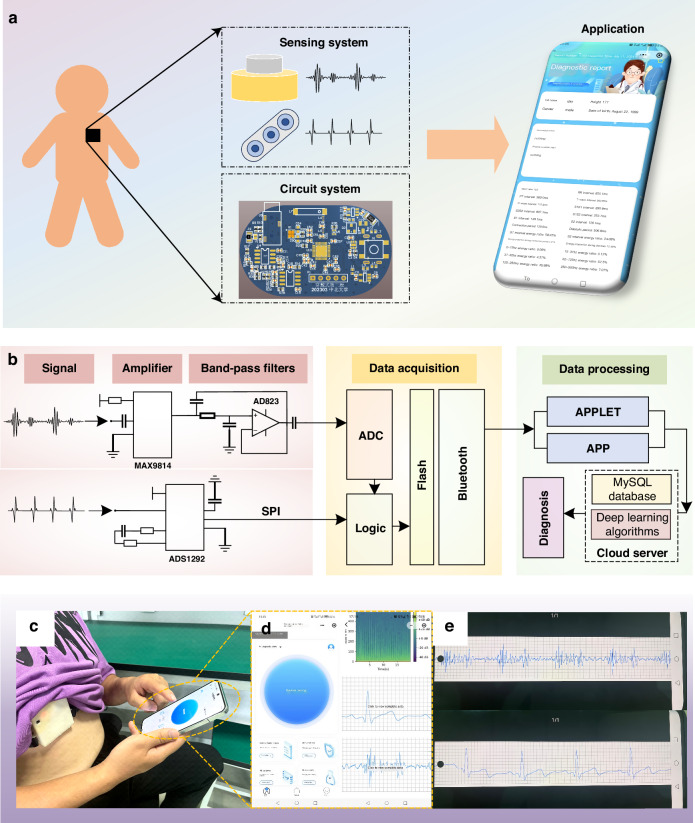
Overall system diagram. **a** The non-invasive intelligent cardiac monitoring system. **b** Schematic diagram of the system, composed of a signal amplifier, band-pass filter, data acquisition, and data processing components. **c** Test diagram for volunteers. **d** Interface diagram of the applet. **e** PCG and ECG signals of the volunteer are displayed on the mobile interface through a custom application

This work introduces an innovative non-invasive cardiac monitoring system that facilitates real-time, synchronous monitoring of human ECG and PCG signals. The system is comprised of three distinct yet interconnected components: a sensing system, a circuit system, and an application. The overall architecture of the system is illustrated in Fig. 1a, offering a comprehensive view of its design and functionality. The sensing system, at the heart of the monitoring system, comprises two key modules: a heart sound module and an electrocardiogram module. These modules work in tandem to capture and transmit both ECG and PCG signals. The heart sound module is designed to detect and record the mechanical vibrations produced by the heart during its contraction and relaxation cycles, while the electrocardiogram module captures the electrical signals that propagate through the heart as it beats.

The sensing system captures feeble PCG and ECG signals, which are prone to being disrupted by ambient noise and other external interferences. To combat this issue, we’ve integrated a circuit system behind the sensing system, as depicted in Fig. 1a. This system employs a compact PCB circuit to refine the analog signals by filtering out unwanted noise and amplifying the signal strength. Once processed, the signals undergo digital conversion facilitated by an ADC converter. These digital signals are then efficiently transmitted to the data management system’s terminal at a rapid 1 kHz sampling rate, utilizing a reliable Bluetooth module for wireless communication. Figure 1b illustrates the process of the circuit system.

The data management system is presented through an application, boasting a frontend interface including functions such as data acquisition, data storage, data processing, efficient querying, and result analysis. The backend architecture is segmented into two distinct facets: the doctor’s portal and the management side. The doctor’s portal empowers medical professionals to remotely scrutinize and diagnose patients’ data. In addition, the management side oversees the comprehensive management of doctors, users, and data, ensuring the system’s smooth operation and data integrity. Figure 1c, d show the resulting interface during the diagnosis process within the application, offering a glimpse into its intuitive design. Additionally, Fig. 1d, e present the application’s interface alongside the data playback and query interface, highlighting its versatility and user-centric design.

### Design and simulation of heart sound sensor

**Fig. 2:**
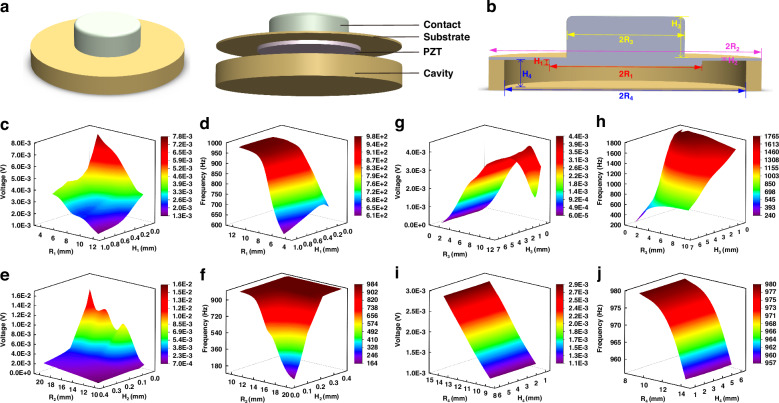
The parameterized simulation results of the heart sound sensing structure. **a** Heart sound sensor structure diagram. **b** Cross-sectional view of the heart sound sensor and the symbolic representation of the parameters of its structures. **c** Influence of PZT structure parameters on the output voltage. **d** Influence of PZT structure parameters on the characteristic frequency. **e** Influence of copper substrate structure parameters on the output voltage. **f** Influence of copper substrate structure parameters on the characteristic frequency. **g** Influence of contact structure parameters on the output voltage. **h** Influence of contact structure parameters on the characteristic frequency. **i** Influence of cavity structure parameters on the output voltage. **j** Influence of cavity structure parameters on the characteristic frequency

The heart sound signal is captured by an advanced contact-type piezoelectric sensor, whose fundamental design is depicted in Fig. 2a. This sensor comprises a contact structure, a piezoelectric sensor structure (consisting of a copper substrate and PZT), and a cavity structure, arranged in a top-to-bottom configuration. The piezoelectric PZT serves as the core sensing element of the heart sound sensor, enabling it to convert mechanical vibrations into electrical signals. When the heart sound signal interacts with the contact structure, it causes the contact to vibrate, leading to deformation within the PZT structure. Due to the direct piezoelectric effect, this deformation generates an electrical signal that is output by the sensor.

Given the inherent weakness of the human heart sound signal, which primarily operates within a frequency range of 20–600 Hz, traditional sensors often struggle to accurately capture and record this vital information. To overcome this challenge and ensure a high-quality collection of human heart sound signals, we employed COMSOL finite element simulation software to simulate and analyze the structural parameters of the heart sound sensor before its integration into the overall system. In the sensing model depicted in Fig. 2a, the contact structure is crafted from rubber, while the base of the piezoelectric sensor and the cavity structure are constructed from copper. Notably, the PZT used is a composite piezoelectric material PZT 1–3, the fabrication process of which is introduced later in the text.

The sensitivity and bandwidth of the sensor are pivotal performance indicators, with sensitivity intimately tied to the output voltage and bandwidth inherently linked to the characteristic frequency. To obtain the optimal structural parameters that would maximize these metrics, we parameterized the simulation study. This analysis contains the copper substrate, PZT structure, cavity structure, and contact structure of the contact-type heart sound sensing structure. Figure 2b illustrates the cross-sectional view of the heart sound sensor as well as the symbolic representation of the structural parameters of its various components. The findings, as illustrated in Fig. 2c–j, reveal the intricate interaction between these structural parameters and their influence on both the output voltage and characteristic frequency.

Figure 2c, d portray the intricate correlation between the radius and thickness of the PZT structure and their influence on both the output voltage and characteristic frequency. In these simulations, the PZT’s radius was varied within a range of [5,12] mm, while its height was adjusted within [0.1,1] mm. Utilizing the COMSOL software for finite element simulations, we uncovered a notable trend: as the radius and thickness of the PZT decrease, the output voltage escalates while the characteristic frequency diminishes, although the thickness exerting a comparatively subtle influence on the latter. Turning our attention to the copper substrate, Fig. 2e, f reveal the parameterized simulation results, indicating that an enlarged radius within [10, 20] mm and a reduced thickness within [0.04,0.4] mm for the copper substrate lead to an increase in output voltage and a decrease in characteristic frequency.

Figures 2g, h and i, j present the results of parameterized simulations conducted on the sensor’s contact structure and cavity structure, respectively. For the contact structure, the simulations explored a range of radii from [1,10] mm and thicknesses from [0.5,6.5] mm. The findings indicate that while the thickness of the contact structure has a negligible impact on the output voltage, the voltage increases with the radius’s enlargement until it reaches a threshold at 10 mm, beyond which it begins to decline. In contrast, the characteristic frequency remains relatively unaffected by the radius but exhibits a positive correlation with decreasing thickness. Turning to the cavity structure, Fig. 2i, j reveal that the sensor’s output voltage scales directly with the radius of the cavity, whereas the characteristic frequency inversely correlates with it. Notably, the height of the cavity structure does not significantly influence the sensor’s performance.

After conducting thorough parameterized simulations and carefully considering the impact of various parameters on the sensor’s overall performance, we have determined the optimal set of parameters. These parameters include: the copper substrate for the Piezoelectric Sensor with a radius of 15 mm and a thickness of 0.12 mm; the PZT with a radius of 9 mm and a thickness of 0.5 mm; the copper cavity structure with a radius of 3 mm and a thickness of 1.2 mm; and the contact structure with a radius of 7 mm and a thickness of 5 mm.

**Fig. 3:**
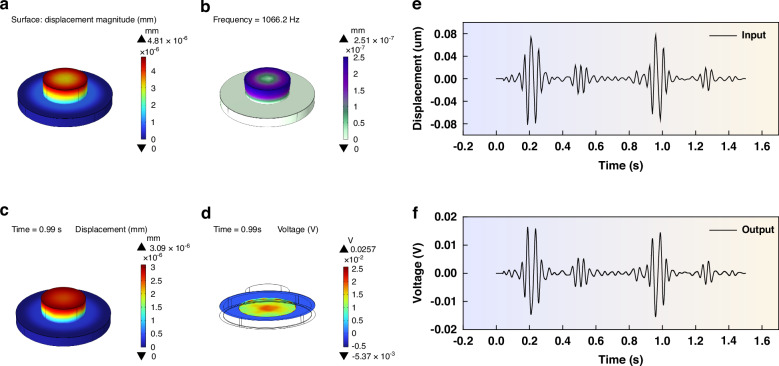
Simulation results of the heart sound sensor. **a** Displacement simulation result of steady-state analysis. **b** Simulation results of characteristic frequency. **c** Displacement results of the transient simulation. **d** Potential results of the transient simulation. **e** Heart sound signal input during the transient simulation. **f** Signal obtained from the transient simulation

Utilizing the optimized parameters as mentioned above, we constructed a sensing model. By applying a pressure of 1 Pa to the upper surface of the single contact structure, we conducted both steady-state and characteristic frequency simulations to evaluate the sensor’s performance. Figure 3a vividly shows the displacement simulation results under steady-state conditions, revealing that the maximum displacement is centered at the contact structure, with a total maximum displacement of 4.81 nm. Furthermore, the characteristic frequency simulation results presented in Fig. 3b indicate that the sensor’s natural frequency of 1066.2 Hz, which aligns perfectly with the frequency range of heart sound signals, thereby validating its suitability for capturing these vital physiological signals.

To confirm the sensor’s performance in capturing heart sound signals, we used the German 3B Scientific human heart and lung sound auscultation model to extract a set of standard heart sound signals, as depicted in Fig. 3e. Subsequently, we input the PCG signal into the COMSOL software, using the Solid Mechanics-Electrostatic physics interface to conduct intricate transient simulations. The simulation results shown in Fig. 3f, highlight the sensor’s remarkable performance, as the contact-type PZT sensing structure efficiently outputs the heart sound signals, thereby confirming its ability to detect this physiological signal. Additionally, Fig. 3c, d present the sensor’s behavior at 0.99 s during the transient simulation process, revealing a maximum displacement of 3.09 nm, and an electric potential distribution that peaks at the center of the PZT, gradually diminishing towards the edges, with the overall maximum electric potential reaching 0.257 mV, further proving the sensor’s sensitivity and precision.

### Fabrication and integration of sensors

**Fig. 4:**
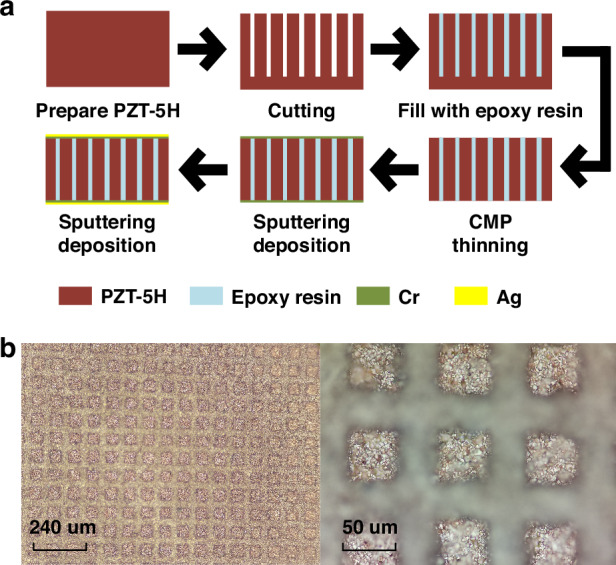
Fabrication of PZT 1–3. **a** Diagram of the Fabrication Process. **b** Morphology of PZT 1–3

Figure 4a shows the preparation process of the PZT1–3 material. The overall procedure begins with the preparation of PZT-5H ceramic material with a thickness of 700 um, which serves as the foundational base for the composite. Utilizing a precision dicing machine (DAD332), the ceramic is cut into a columnar array, with each column having a cross-sectional side length of 50 um and a spacing of 30 um between the columns. A height of 200 um is retained at the base of the ceramic to ensure the stability of the PZT structure during the subsequent pouring stage. Next, the cut PZT ceramic is carefully placed within a specialized mold, where an epoxy resin solution is poured. To ensure the solution’s purity and absence of bubbles, it is subjected to vacuum treatment within a vacuum drying chamber. Once the solution has thoroughly cured, an LP50 thinning polishing machine is employed to remove the excess epoxy resin and the 200 um-thick supporting material at the bottom. Finally, the composite PZT material undergoes sputtering deposition processes, resulting in the formation of the 5 um thick Cr and 10 um thick Ag layers on both its upper and lower surfaces. The Cr layer serves a dual purpose: it enhances the adhesion between the Ag layer and the substrate, ensuring a reliable connection, while acting as a barrier to prevent any reactions that may arise from the direct contact between Ag and the substrate. The Ag layer, functions primarily as the electrode layer, facilitating the efficient transmission of electrical signals. Figure 4b presents a detailed view of the morphology of the processed PZT1–3 composite material, captured using an optical microscope. At these resolutions, it becomes evident that the surface of the PZT1–3 material is exceptionally clear and the structure remains undamaged, showing the precision and quality of the processing techniques employed.

Following the successful preparation of the PZT1–3 composite piezoelectric material, we used simulation results to customize the copper substrate for the piezoelectric sensor. The copper substrate’s surface was polished to ensure optimal adhesion and a specialized SL3334 ceramic adhesive was employed to bond the PZT1–3 material to the substrate. After allowing the adhesive to cure thoroughly for 24 h, the preparation of the PZT sensor was deemed complete.

**Fig. 5:**
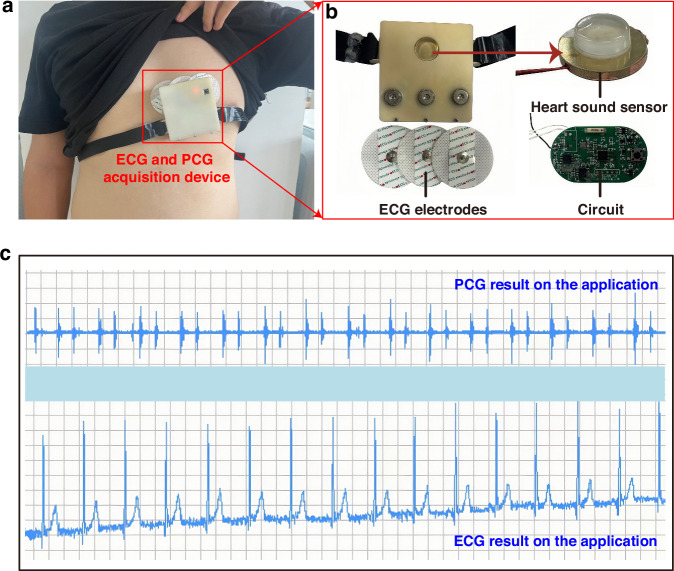
Integration and testing of the sensor. **a** Schematic diagram of wearing the ECG and PCG synchronous acquisition device. **b** Actual images of internal components. **c** Display of ECG and PCG signals on the application interface in the lying down position

Then, we utilized computer numerical control (CNC) machining to create a copper cavity structure. This cavity was integrated with the PZT sensor’s copper substrate through advanced thermal bonding technology, ensuring a robust and reliable connection. To complete the sensing structure, a custom-designed rubber elastic contact structure was carefully bonded to the surface of the piezoelectric sensor’s copper substrate using a high-performance adhesive. This arrangement positions the elastic contact structure and the PZT1–3 composite material on opposite sides of the copper substrate, with the PZT1–3 material nestled securely within the cavity space. Figure 5b illustrates the completed PZT contact-type heart sound sensing structure.

Furthermore, we have opted to utilize the widely available ECG electrodes prevalent in the market to capture human ECG signals. Among the two standard ECG methods, namely the twelve-lead ECG and the three-lead ECG, we have carefully considered their respective merits. While the twelve-lead ECG undoubtedly offers a more comprehensive insight into cardiac electrical activity, it falls short in terms of portability and suitability for wearable designs. In contrast, the three-lead ECG, with its electrodes typically positioned on the right arm, left arm, and left leg, provides a reliable and efficient means of capturing heart activity. To further enhance wearability and portability, we have devised an innovative electrode layout, as depicted in Fig. 5b. This layout features three equidistant and collinear electrodes strategically placed over the heart area, complemented by the incorporation of electrode buckles. This design not only ensures accurate ECG signal acquisition but also eliminates the constraints posed by traditional lead wires, significantly enhancing user comfort and convenience.

Ultimately, the heart sound sensor, three-lead ECG electrodes, and the hardware acquisition system have been integrated to create a synchronous PCG and ECG acquisition device, as shown in Fig. 5a. The ECG electrodes, embodying a practical and wearable solution, are complemented by a designed strap that ensures a comfortable and secure fit between the heart sound sensor and the user’s body, facilitating accurate and reliable data acquisition.

## Results and discussion

### Analysis of human body ECG and PCG signals

To validate the practical efficacy and applicability of our system, we conducted tests on a healthy male volunteer aged 25. As depicted in Fig. 5a, we utilized the wearable device proposed in this paper, which is designed for the simultaneous acquisition of ECG and PCG signals. Using the developed mobile application, we captured the ECG and PCG signals of the volunteer in real time, ensuring the data acquisition process. To guarantee the accuracy of our testing, we positioned the volunteer in a supine, resting state and collected the signals for 14 s. This approach aimed to minimize any potential interferences that could compromise the quality of the recorded signals. Upon completion of the data acquisition phase, we swiftly processed the collected signals and extracted the test results. As illustrated in Fig. 5c, these results were presented within the mini-program’s interface, offering an overview of the ECG and PCG signals captured during the testing session.

**Fig. 6:**
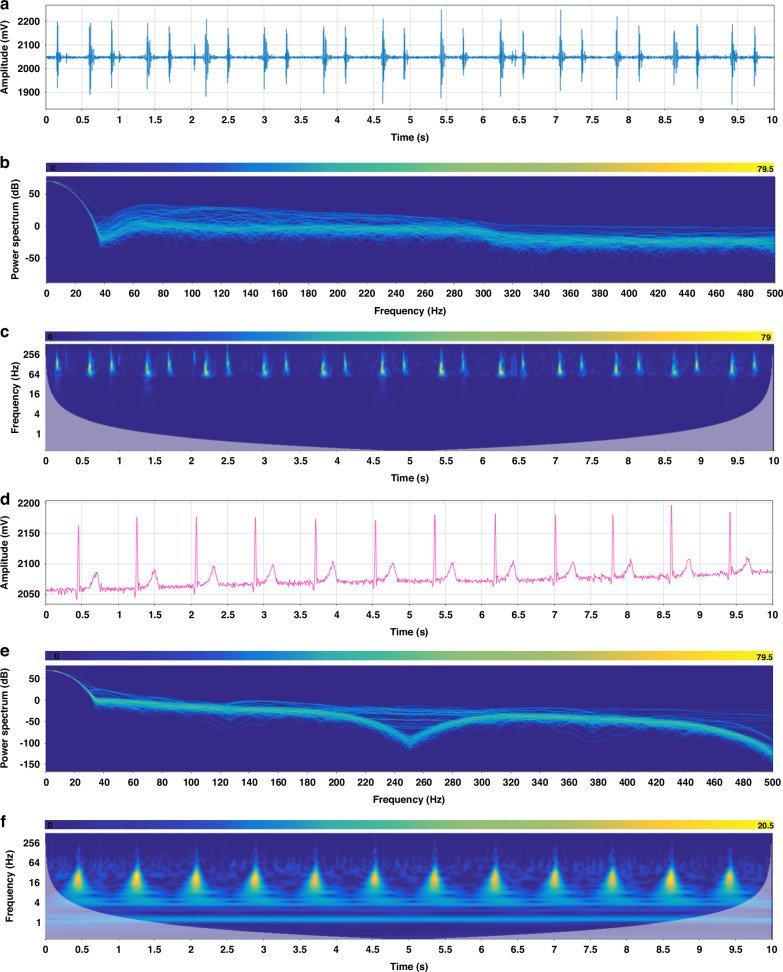
Signal analysis of ECG and PCG within 0–10 s (participant in supine position). **a** PCG signal. **b** Spectrum of PCG signal. **c** Spectrogram of PCG signal. **d** ECG signal. **e** Spectrum of ECG signal. **f** Spectrogram of ECG signal

We have extracted the PCG and ECG signals from the initial 10 s of the recorded data in Fig. 5c, and the time-domain representations of these signals are presented in Fig. 6a, d, respectively, providing a clear visualization of their waveforms. To gain further insights into the frequency content of these signals, we performed a Fast Fourier Transform (FFT) analysis, resulting in the spectrums shown in Fig. 6b, e. These spectrums reveal that the frequencies of both the PCG and ECG signals exhibit temporal variations. Moving on to the power spectrum diagrams, Fig. 6c, f present an assessment of the energy distribution within the PCG and ECG signals, respectively. In Fig. 6c, the first heart sound (S1) and the second heart sound (S2) are prominently displayed, with S1 exhibiting a higher energy value compared to S2. This observation highlights the distinctiveness of these two cardiac sounds in terms of their energetic properties. Furthermore, Fig. 6f provides a detailed view of the ECG signal, clearly revealing the characteristic QRS-T waves. Additionally, by analyzing the frequency distributions, we can observe that the heart sound signal primarily resides within the 60–200 Hz range, while the ECG signal is concentrated within the 4–60 Hz band. These findings are consistent with the known frequency distributions of ECG and PCG signals, thereby validating the accuracy and reliability of our signal processing and analysis methods.

### Stability testing of ECG and PCG synchronous acquisition system

**Fig. 7:**
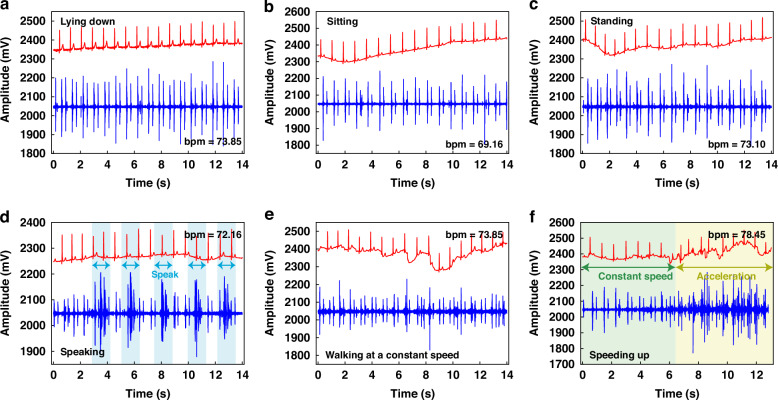
Test results under different conditions. **a** Test results of the subjects in lying down. **b** Test results of the subjects in sitting. **c** Test results of the subjects in standing. **d** Test results of the subjects in speaking. **e** Test results of the subjects in steady walking. **f** Test results of the subjects in accelerated walking states

We further employed the wearable device to assess the ECG and PCG signals of a male volunteer under varying physiological states. As depicted in Fig. 7a–f, the volunteer underwent a series of activities, including lying down, sitting, standing, speaking, steady walking, and accelerated walking, while their ECG and PCG signals were continuously monitored.

In analyzing the heart sound signals, we consistently observed the presence of the first heart sound (S1) and the second heart sound (S2) across all three static states (lying down, sitting, and standing), as depicted in Fig. 7a–c. This consistency underlines the reliability of our sensing system in detecting these critical cardiac sounds, regardless of the volunteer’s posture. Turning our attention to the ECG signals, we found that the lying down position created the most optimal results, characterized by minimal baseline drift. In contrast, the sitting and standing positions exhibited slightly higher levels of baseline drift, which could potentially be attributed to the electrical signals generated by muscle movements associated with these postures. Nonetheless, the ECG signals remained clear and discernible, highlighting the robustness of our system in capturing these vital electrical signals under various conditions.

When examining the test results obtained during non-stationary states, it becomes evident that the overall signal quality exhibits a slight decline compared to the static conditions. Specifically, as shown in Fig. 7d, when the tester periodically uttered the phrase “heart sound,” the ECG signal remained largely unaffected. However, the PCG signal exhibited an increase in noise levels during the tester’s vocalization, which subsided upon completion of the vocalization, highlighting the heart sound sensor’s great recovery performance and exceptional sensitivity.

During steady walking, as depicted in Fig. 7e, the ECG baseline drift became more pronounced, while the S1 and S2 heart sound signals remained visible, although with increased noise levels. This observation underlines the challenges posed by dynamic activities on the acquisition of high-quality cardiac signals. The diagram in Fig. 7f shows the results obtained during steady walking followed by accelerated walking. During steady walking, clear S1 and S2 heart sound signals were discernible, whereas during acceleration, significant noise and ECG drift were observed.

In summary, the system has demonstrated remarkable stability in detecting both ECG and PCG signals across various conditions. Notably, while the heart rate varied slightly between 73.00 during most states and 78.45 during accelerated movement, with 69.16 recorded in a sitting position, all measurements remained within normal physiological ranges. To reduce the issue of baseline drift observed in ECG signals, the implementation of a Butterworth band-pass filtering algorithm presents a viable solution. Overall, these experimental findings serve as a testament to the robustness and suitability of the wearable ECG and PCG detection device for daily cardiac monitoring.

### Signal-to-noise ratio analysis of ECG and PCG Signals

The signal-to-noise ratio (SNR) serves as a critical metric for evaluating the performance of sensors in capturing meaningful data. In the context of our study, we have calculated the SNR for both the PCG and ECG signals, drawing upon the results obtained from the stationary states depicted in Fig. 7. For the PCG signal, we have adopted a straightforward approach to calculate the SNR, focusing on the amplitude of the signal itself. This method, outlined in Eq. 1, involves comparing the amplitude of the signal (Vss) to the amplitude of the noise (Vnn), thereby providing a quantitative assessment of the signal.

1 $$SNR=20\ast \,\log \left(\frac{Vss}{Vnn}\right)$$

Table 1 offers an illustration of the amplitude of the signal and noise in three distinct static states: lying down, sitting, and standing. By carefully calculating the SNR for each of these states, we have determined that the SNR is 30.37 dB in the lying down position, 30.75 dB in the sitting position, and 29.00 dB in the standing position. When averaged across these three states, the resulting SNR of the PZT heart sound sensor, which is based on the innovative contact-point structure, stands at a robust 30.04 dB.

**Table 1:** The SNR comparison of ECG and PCG signals

Type of sensor		SNR of PCG signal [dB]			SNR of ECG signal [dB]		
Our sensors	The state of the human body	Vss	Vnn	SNR	Ps	Pn	SNR
	Lying down	330	10	30.37	4350000	163.95	44.23
	Sitting	276	8	30.75	4240000	163.76	44.13
	Standing	340	12	29.00	3680000	145.64	44.03
	Average	30.04			44.13		
Han Seok Cho’s sensors 22		28.00			34.00		
Pengcheng Shi’s sensors 21		25.60			—		
Yirui Li’s sensor 23		27.38			—		
Yuhua Yang’s sensor 11		27.05			—		

On the other hand, for the ECG signal, we have opted to calculate the SNR based on the signal’s power. Equation 2 outlines the methodology for determining the SNR of the ECG signal. In this equation, Ps represents the effective power of the signal itself and Pn indicates the effective power of the noise.

2 $$SNR=10\ast l{\rm{og}}\left(\frac{Ps}{Pn}\right)$$

To accurately calculate the power of the ECG signal, we used the powerful capabilities of MATLAB, ensuring that our results are both reliable and precise. The results of our SNR calculations are presented in Table 1, showing the SNR values obtained in three distinct static states: lying down, sitting, and standing. These values are 44.23 dB, 44.13 dB, and 44.03 dB respectively. Upon averaging these values, we get an overall SNR of 44.13 dB for the ECG signal.

Table 1 presents a comparative analysis of the SNR levels achieved by various ECG and PCG devices. Specifically, we have compared the performance of our sensor with two other synchronized detection instruments. Firstly, the microphone-electrode sensor proposed by Han Seok Cho and his colleagues demonstrates an SNR of 28.00 dB for the PCG signal and 34.00 dB for the ECG signal^22^. Furthermore, the MEMS heart sound sensor-electrocardiogram detector introduced by Pengcheng Shi and his team has an SNR of 25.60 dB for the heart sound sensor^21^. However, it is important to note that the SNR level for the ECG signal was not provided in their study. In contrast, the sensor proposed in this paper surpasses the aforementioned devices in terms of SNR levels for both the ECG and PCG signals. Our sensor achieves a higher SNR of 44.13 dB for the ECG signal and 30.04 dB for the PCG signal, emphasizing its superior performance in capturing cardiac signals. Additionally, compared to the heart sound sensors proposed by Yirui Li^23^ and Yuhua Yang^11^, the heart sound sensor presented in this paper also has a higher signal-to-noise ratio.

In addition, according to ref.^24^, the overall classification accuracy of intelligent diagnosis is 94.21% when the SNR is 10 dB and 95.50% when the SNR is 20.00 dB. The relationship between the SNR and the classification accuracy points out that there is a positive linear relationship between the SNR and the classification accuracy below 25.00 dB. At the same time, ref.^25^ investigates the performance difference between the noise-filtered signal and the unfiltered signal in distinguishing patients with coronary artery disease (CAD) from those without. The study demonstrates that signals containing noise ultimately result in a decrease in the accuracy of CAD classification. Reference^26^ shows that noise above 250 Hz is significant for CAD diagnosis and recognition. It has an extreme sensitivity, and the Area Under the Receiver Operating Characteristic (AUC) index drops significantly. Therefore, improving the SNR of the sensor and reducing the noise have great practical significance on the accuracy of intelligent diagnosis and the stability of the instrument system.

### Joint feature analysis of ECG and PCG Signals

**Fig. 8:**
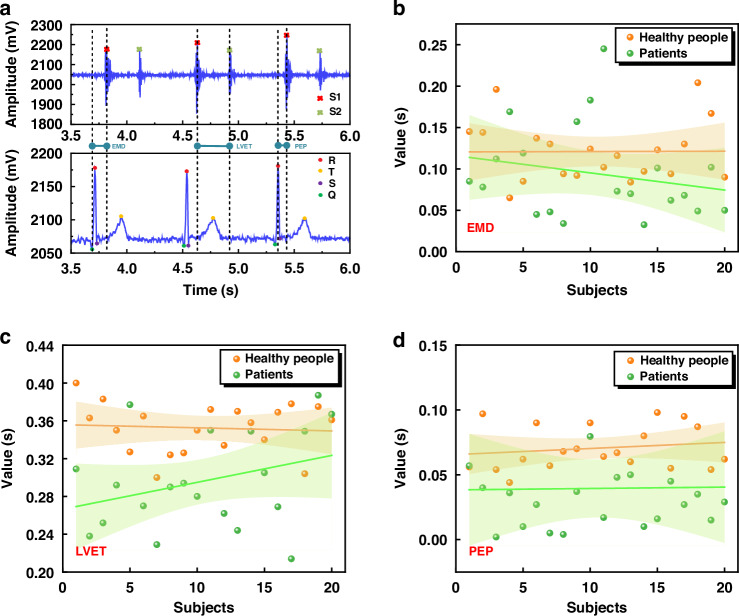
Joint analysis of ECG and PCG signals. **a** Schematic diagram of joint features. **b** Scatter plot of EMD feature parameters, linear regression line, and corresponding 95% confidence interval. **c** Scatter plot of LVET feature parameters, linear regression line, and corresponding 95% confidence interval. **d** Scatter plot of PEP feature parameters, linear regression line, and corresponding 95% confidence interval

Using the wearable ECG and PCG synchronous detection device introduced in this paper, we have successfully captured the human body’s ECG and PCG signals, which serve as the cornerstone for our subsequent feature analysis aimed at intelligent monitoring and early warning of cardiovascular diseases. As the field of cardiovascular research continues to evolve, numerous scholars are actively researching the various impact parameters associated with these diseases, including electromechanical delay time (EMD), left ventricular ejection time (LVET), and pre-ejection period (PEP)^27–29^. These parameters hold significant value in assessing the heart’s contractile function and pumping capacity. Specifically, EMD offers insights into the temporal delay between the heart’s electrical activity and its subsequent mechanical motion, while PEP and LVET provide quantitative measures of the heart’s pumping function. A shorter PEP is indicative of robust cardiac contractile function, whereas an extended LVET may point towards reduced cardiac pumping efficiency. Drawing upon these parameters, we have conducted a joint analysis of the collected signals. To illustrate this process, we take the signals recorded in the lying down state, as depicted in Fig. 7a, as our case study. Figure 8a presents a schematic diagram of the characteristic parameters extracted from these signals.

To start our analysis, we first identify the key points of the ECG signal, namely the Q, R, S, and T points, as well as the S1 and S2 positions within the PCG signal. Next, we compare the temporal relationships between the ECG and PCG signals, specifically focusing on three critical parameters: EMD, LVET, and PEP. To obtain these parameters, we calculate the time difference between a 14 s waveform segment shown in Fig. 5c. For EMD, we calculate the time from the onset of the QRS complex in the ECG to the peak of the S1 in the PCG. Similarly, LVET is determined by measuring the time between the first heart sound (S1) and the second heart sound (S2) in the PCG signal, while PEP is the time from the peak of the R wave in the ECG to the peak of the S1 in the PCG. By averaging these measurements across the analyzed waveform segment, we get the following values: EMD is 0.094 s, LVET is 0.297 s, and PEP is 0.069 s. These quantitative results offer a basis for the assessment of cardiac function and potential early detection of cardiovascular diseases.

Building upon our initial findings, we have expanded our study by collecting a dataset comprising 40 cases of ECG and PCG data, equally divided between 20 healthy subjects and 20 patients diagnosed with coronary heart disease. It is crucial to note that the healthy subject data was sourced from volunteers within the school community, whereas the patient data was obtained from the Department of Cardiology at the Second Affiliated Hospital of Shanxi Medical University. The study was approved by the Second Affiliated Hospital of Shanxi Medical University and North University of China. The data collection process was standardized according to the “Shanxi Medical University Scientific Research Ethics Review Committee Regulations” and the “North University of China Scientific Research Ethics Review Committee Regulations.” The collected data were used as research samples, and all subjects were informed and consent was obtained.

Figure 8b–d present a detailed analysis of the characteristic parameters EMD, LVET, and PEP, respectively, through scatter plots that distinctly differentiate between healthy individuals (represented by yellow dots) and patients with coronary heart disease (green dots). To gain further insights, we have conducted linear fitting on these scatter plots, revealing distinct linear trends and 95% confidence intervals for both healthy and patient populations.

These graphics reveal a consistent pattern: for EMD, LVET, and PEP, the fitted lines for healthy individuals are consistently positioned above those of coronary heart disease patients. Moreover, the 95% confidence intervals for patients are notably wider than those of healthy individuals, indicating a greater dispersion in the values of these parameters among patients. Specifically, our analysis of 40 cases indicates that healthy individuals typically exhibit EMD values ranging from 0.1 s to 0.15 s, whereas patients with coronary heart disease consistently display lower EMD values, falling below 0.1 s. Similarly, LVET values for healthy individuals cluster within the 0.32 s to 0.4 s range, whereas patients show a narrower range of 0.24 s to 0.32 s. For PEP, healthy individuals have measurements within 0.05 s to 0.1 s, whereas patients exhibit values below 0.05 s.

These findings underline the potential of the combined ECG and PCG signals as an assessment tool for cardiac health. As the dataset continues to grow, deeper analyses of feature values such as EMD, LVET, and PEP can reveal valuable insights into the evolution of coronary heart disease, facilitating intelligent diagnosis and prognosis, and providing crucial information to support clinical decision-making by healthcare professionals.

## Conclusions

In pursuit of realizing digital diagnosis of cardiovascular diseases and developing portable detection devices, we introduce a wearable ECG and PCG synchronous detection system. A contact-type heart sound sensing structure based on PZT1–3 composite piezoelectric material is prepared, achieving a high-quality acquisition of human PCG signals. Rigorous testing has confirmed the system’s remarkable stability across various operating conditions, while significantly enhancing the SNR of ECG and PCG signals to levels of 44.13 dB and 30.04 dB, respectively. This work represents a significant advancement in cardiovascular disease screening technology, providing new avenues for early diagnosis and treatment. Furthermore, we have conducted a joint analysis of ECG and PCG signals, extracting critical parameters such as EMD, LVET, and PEP, and performing in-depth feature analysis on a dataset comprising 40 cases, revealing the differences between healthy individuals and patients with coronary heart disease in the three joint feature parameters. Looking ahead, we plan to use deep learning algorithms, grounded in the insights gained from this research, for the classification and prediction of cardiovascular diseases. This endeavor holds immense potential, offering novel ideas and methodologies for the future of intelligent diagnosis in the field of cardiovascular medicine.
